# Associations of lifestyle with burnout risk and recovery need in Flemish secondary schoolteachers: a cross-sectional study

**DOI:** 10.1038/s41598-024-53044-w

**Published:** 2024-02-08

**Authors:** Yanni Verhavert, Tom Deliens, Jelle Van Cauwenberg, Elke Van Hoof, Christophe Matthys, Juriena de Vries, Peter Clarys, Kristine De Martelaer, Evert Zinzen

**Affiliations:** 1 Department of Movement and Sport Sciences, Vrije Universiteit Brussel, Pleinlaan 2, 1050 Brussels, Belgium; 2 Department of Public Health and Primary Care, Faculty of Medicine and Health Sciences, Ghent University, Corneel Heymanslaan 10, 9000 Ghent, Belgium; 3 Research Foundation – Flanders (FWO), Leuvenseweg 38, 1000 Brussels, Belgium; 4 Brussels, Belgium; 5 Department of Endocrinology, University Hospitals Leuven, Herestraat 49, 3000 Leuven, Belgium; 6 Clinical and Experimental Endocrinology, Department of Chronic Diseases and Metabolism, KU Leuven, Herestraat 49, 3000 Leuven, Belgium; 7 Department of Health, Medical and Neuropyschology, University of Leiden, Wassenaarseweg 52, 2333 AK Leiden, The Netherlands

**Keywords:** Diseases, Health care, Risk factors

## Abstract

Teacher burnout and high recovery need are a topic of concern for educational institutions. This cross-sectional study assesses the association between lifestyle (including physical activity (PA), sedentary behavior (SB), dietary behavior and sleep), burnout risk and recovery need in 1878 secondary schoolteachers in Flanders. In September–October 2019, an online-questionnaire assessing burnout (i.e., emotional exhaustion, depersonalization, personal accomplishment), recovery need, PA-domains, SB-domains, dietary behavior (including fruit and vegetable intake and diet quality) and sleep during the week and the weekend was completed. Multiple linear regression models were applied. More emotional exhaustion was associated with more domestic and garden PA, work-related SB and sleep during the weekend, whereas higher scores of emotional exhaustion were associated with lower fruit intake, diet quality and less sleep during the week. More depersonalization was associated with more leisure-time PA and work-related SB and with lower fruit intake. Teachers showing more recovery need, showed more domestic and garden PA and work-related SB, but less leisure-time PA and sleep during the week. Future research should use longitudinal or experimental designs to get more insight into causality. Despite the low effect sizes, education networks and schools might benefit from promoting and facilitating a healthy lifestyle in secondary schoolteachers.

## Introduction

Burnout has been defined as a “prolonged response to chronic emotional and interpersonal stressors on the job”^1^ and represents a major concern in today’s workplaces^2^. Individuals suffering from burnout report three main problems. They report being emotionally extended (i.e., emotional exhaustion), and have a negative evaluation of their recipients (i.e., depersonalization) and their work performance (i.e., reduced personal accomplishment)^1^. A recent report about the prevalence of burnout in Belgium indicated that, in 2019, 13.6% of the employees reported burnout symptoms compared to 10.2% in 2004^3^. In addition, between 2016 and 2020, the number of Belgian employees receiving disability benefits for long-term illness due to burnout increased by 33%^3^. In 2019, almost half a billion EUROs were spent on long-term disability due to burnout in Belgium, which is an increase of 22% since 2016^3^. The global cost of burnout and stress is estimated at more than USD 300 billion every year^4,5^. Besides its financial impact, burnout is found to be a predictor of several unfavorable physical, psychological and professional outcomes such as cardiovascular diseases, headaches, gastrointestinal issues, insomnia, depressive symptoms, job dissatisfaction and absenteeism^6^.

It has been shown that the development of burnout is a result of prolonged exposure to work-related stressors, such as work overload, role conflict and ambiguity, work-home conflict, compensation inequity, job insecurity, job complexity and an imbalance between demands and resources at work^7–9^. However, research also showed that work-related stressors and burnout are only moderately correlated, and that recovery after/outside working hours (i.e., the ability to reverse short-term physiological or psychological stress responses to work stressors during leisure time) is an important moderator of the stressor-burnout relationship^7^. When insufficient recovery occurs for a long time, individual’s physiological stress systems will be constantly activated, leading to an even higher need for recovery, feelings of fatigue, poor sleep and eventually burnout^7,10–12^. Therefore, it can be said that recovery need and burnout are closely related, and that recovery need should be taken into account when conducting research on burnout, as it is considered a precursor of burnout^10^.

In the past, it was said that burnout mainly occurs in employees doing ‘people work’, including jobs that require the provision of care or support^13^. Nowadays, it is clear that its occurrence has broadened to employees doing all kinds of jobs. As teaching is recognized as a highly stressful job, teachers are being considered as an at-risk population for burnout^8^. In 2021, a report about sick leave in Flemish secondary schoolteachers showed an average sick leave of 15 days per person per year. More specifically, psychosocial diseases were accountable for 38% (in women) and 43% (in men) of the sick leave among Flemish primary and secondary schoolteachers (numbers for secondary schoolteachers alone were not available)^14^. The main stressors of a teacher’s job include, among other things, decreased autonomy, high workload (including teaching and administrative tasks), increased number of students, misbehavior of students and low support of supervisors^15–19^. Furthermore, teacher burnout is leading to decreased student–teacher interaction, decreased ability in dealing with classroom behavior problems, lower quality of student motivation and decreased class average of students’ school grades^20–22^. Due to the negative consequences on occupational health and educational outcomes, teacher burnout is an increasing concern for educational institutions. Accordingly, it is of utmost importance to investigate how burnout in teachers can be managed and preferably prevented.

Lifestyle (interventions) may play an important role in enhancing recovery and in preventing and/or managing teacher burnout, as several studies demonstrated that different lifestyle factors (e.g., physical activity (PA), sedentary behavior, diet, sleep) are associated with mental health^23–27^. A systematic review by Lopresti, Hood^28^ recommended future research to be directed towards a better understanding of the possible role of PA, diet and sleep in mental health conditions. The general idea behind the lifestyle-burnout associations is that a healthy lifestyle results in physiological (e.g., better executive functioning) and psychological (e.g., better mood) adaptations that promote recovery from work and increase one's capability to handle work-related stressors (see e.g., Verhavert, De Martelaer^29^ and de Vries and Bakker^30^ for overviews of proposed underlying mechanisms). For example, PA can decrease burnout risk as it helps to distract from work-related problems and increases stress resilience by uplifting mood^31,32^. On the other hand, elevated levels of sedentary behavior may lead to decreased mood and a reduction in motivation for and active involvement in rewarding activities^33^. Burnout risk may also be influenced by one's diet, as an unhealthy diet may result in decreased mood^34^ and a higher intake of fruits and vegetables is associated with enhanced self-efficacy and reduced psychological distress^35^. Moreover, research showed that brain-derived neurotrophic factor (BDNF-) levels, which are decreased in people with burnout symptoms^36^, may be influenced by dietary intake^37,38^. Lastly, during sleep, important physiological restoration processes take place, which help to (re)build energetic resources^39^.

A recent systematic review by Verhavert, De Martelaer^29^, investigating the association between burnout (risk) and PA, sedentary behavior (SB) and dietary behavior, concluded that sufficient PA may indeed be effective in reducing burnout risk. It was found that being physically active two to five times per week for 20 to 60 min during six to 18 weeks resulted in reductions in burnout risk and/or its dimensions ranging from 6.9 to 41.3%. Although de Vries and Bakker^30^ recommended that a distinction between different domain-specific PA (i.e., during transport, household chores, gardening and leisure-time) is relevant for predicting burnout, only one study included in the latter review investigated the effect of PA performed in a specific domain (i.e., work-related PA) on all burnout dimension levels^30^. This is unfortunate, as the voluntary and pleasurable character of leisure-time PA is likely most beneficial to reduce burnout risk compared to work-related PA, which is often less voluntary and pleasurable^30^. Furthermore, only two observational studies regarding SB and two observational studies concerning dietary behavior were included in the review of Verhavert, De Martelaer^29^, showing worse burnout dimension levels and/or higher burnout risk when being more sedentary and eating unhealthier. These observational studies showed some methodological shortcomings (e.g., no use of validated questionnaires), making it difficult to draw reliable conclusions regarding the relationships of sedentary and dietary behavior with burnout risk. Although it is shown that a high fruit and vegetable intake as well as a good overall diet quality are recommended given their health-promoting properties^40^ and given their evidenced link with other mental outcomes (e.g., an inverse association with depression and anxiety^41^), studies assessing the link between fruit and vegetable intake, diet quality, or other dietary intakes or behavior and burnout risk and/or recovery need are limited. Moreover, the available but limited research on this specific topic is equivocal. A recent study by Penttinen, Virtanen^42^ showed that frequent consumption of healthy food items was associated with fewer burnout symptoms, whereas Chui, Bryant^43^ found that more fast food and less fruit and vegetable intake were associated with lower emotional exhaustion and higher depersonalization. It should be mentioned though, that comparison of existing literature is difficult due to different assessment and analysis methods of dietary intake and burnout. Unfortunately, none of the included studies in the systematic review of Verhavert, De Martelaer^29^ used a holistic approach (i.e., investigating the relationship between burnout risk and PA, SB and dietary behavior as a whole). Besides these lifestyle factors, sleep may also play a valuable role in enhancing recovery and in preventing and/or managing burnout. Unfortunately, contradicting results regarding the relationship of burnout with this lifestyle factor exist. Soderstrom, Jeding^44^, for example, found that too little sleep (i.e., less than six hours per night) in employees of an IT company was a main risk factor for burnout development, whereas Metlaine, Sauvet^45^ demonstrated that total sleeping time and burnout were not significantly associated in white-collar workers. In addition, a recent review concluded that a negative cross-sectional association between sleep quality and burnout exists^46^. It should be mentioned that most of the included studies were conducted in health professions^46^. Importantly, the findings of the few included longitudinal studies were mixed, showing either no or negative associations between sleep and burnout^46^.

To the best of our knowledge, no previous studies investigated the associations between different lifestyle factors (including PA, SB, dietary behavior and sleep) and burnout risk and recovery need in schoolteachers. Therefore, the aim of this study was to analyze the associations between lifestyle factors (including PA domains, SB domains, dietary behavior and sleep) and burnout dimensions as well as recovery need in schoolteachers. As teachers are at high risk for burnout, we focused on Flemish secondary schoolteachers as the specific population of interest. We hypothesize significant associations between all included lifestyle factors and the burnout dimensions and recovery need. We expect that the directions of these associations will depend on the specific behaviors considered, namely that more transport-related PA, domestic and garden PA, leisure-time PA, fruit- and vegetable intake, diet quality and sleep are negatively related to burnout risk and recovery need, whereas work-related PA and SB across all SB-domains are expected to be positively related to burnout risk and recovery need.

## Methodology

### Design and participants

In this cross-sectional study, a non-probability cluster sampling strategy was used to recruit Flemish secondary schoolteachers. All 1075 secondary schools in Flanders, the Dutch-speaking part of Belgium, were contacted through e-mail and telephone, and were asked to send an e-mail with a link to our online questionnaire to their entire teaching staff. The Flemish Department of Education as well as all educational networks (i.e., schools owned by the Flemish community, subsidized public schools and subsidised free schools) were asked to promote the study among the secondary school principals. We also visited a convenient selection of 43 schools (with a good geographical spread) to promote our study face-to-face. The link to the online questionnaire was also spread through social media (e.g., Facebook, Twitter) and advertisements via diverse communication channels of the Flemish Department of Education (e.g., electronic newsletters). Flemish secondary school teachers between 21 and 65 years old, speaking the Dutch language and willing to give informed consent were included in the study, whereas teachers being in sick leave due to illness (except for burnout), teachers not being able to be physically active due to injuries and/or teachers with an adapted dietary pattern (due to allergies or following a diet) or non-teaching employees working in secondary schools (e.g., administrative staff and principals) were excluded from the study. An a priori sample size calculation was conducted using the statistical software ‘GPower 3.1’. A sample size analysis was performed based on the t-tests family for a linear multiple regression analysis (fixed model, single regression coefficient), with alpha = 0.05, power 0.8, effect size of f^2^ = 0.02 and 16 predictors. The relatively low effect size was based on previous studies^47,48^. The estimated total sample size (n) needed was 395.

### Procedure and measurements

In Flanders, school years begin on the 1st of September and last until the 30th of June. Teachers were asked to complete an online questionnaire during the last week of September and the first week of October 2019. During these two weeks, two reminders were sent to all secondary schools, on the fourth and eighth day after activation of the online questionnaire. The online questionnaire was developed by using the web-based software Qualtrics (Qualtrics, Provo, UT) and consisted of questions regarding burnout and recovery need, PA, SB, dietary behavior and sleep, alongside socio-demographics (i.e., age, sex, marital status, number of children (if any), ethnicity, diploma), anthropometrics (i.e., self-reported height and weight; which show good to very good reliability and validity compared to objective measurements^49–51^) and work-related factors (e.g., questions regarding weekly amount of teaching and in which school(s) they were working). The study protocol was approved by the Medical Ethics Committee of the University Hospital (UZ Brussel, Brussels, Belgium; B.U.N. 143201940533). Research was performed in accordance with relevant guidelines/regulations and all participants gave their informed consent prior to participating in the study.

#### Burnout risk

Risk of burnout was assessed using the Utrecht Burnout Scale for teachers (UBOS-L)^52^, which is based on the internationally recognized and validated Maslach Burnout Inventory (MBI)^53^. The UBOS-L questionnaire is the Dutch version of the original MBI and was especially developed for teachers. The survey assesses three dimensions of burnout, namely emotional exhaustion (eight items, e.g., “I feel mentally exhausted because of my job), depersonalization (seven items, e.g., “I don't really care what happens to some students”), and personal accomplishment (seven items, e.g., “I deal very effectively with the problems of my students”). Items were answered on a seven-point Likert scale ranging from 0 (i.e., never) to 6 (i.e., every day). An average score on each dimension was calculated (Emotional exhaustion: higher scores = feeling more emotionally exhausted, depersonalization: higher scores = feeling more cynical about their job, personal accomplishment: higher scores = feeling more competent at work). These three average dimension scores were then combined to estimate burnout risk. Individuals scoring high on emotional exhaustion (i.e., > 2.5) and low on personal accomplishment (i.e., < 3.56), or high on emotional exhaustion (i.e., > 2.5) and high on depersonalization (i.e., > 1.43 for men and > 2.00 for women), are considered at risk for burnout based on the UBOS-L norms^52^. This validated questionnaire has very good to excellent psychometric properties^52^. Schaufeli and van Dierendonck^52^ reported acceptable to good internal consistency across dimensions: emotional exhaustion: α = 0.91, depersonalisation: α = 0.73, personal accomplishment: α = 0.85. Based on our data, the following acceptable to good internal consistencies were found: emotional exhaustion: α = 0.89, depersonalisation: α = 0.70, personal accomplishment: α = 0.80.

#### Recovery need

Recovery need was assessed by means of the validated Short Inventory to Monitor Psychosocial Hazards (SIMPH)^54^. Only the ‘recovery need’ part of this particular questionnaire was used, including five items (e.g., “I find it difficult to relax at the end of a working day”) with a dichotomous response scale (i.e., yes (= 1) or no (= 0)). A total score was calculated by summing the item scores. Participants having a score ≥ 3 were classified as having a ‘high need for recovery’. The ‘recovery need’ part of the SIMPH has good psychometric properties and shows an acceptable internal consistency (α = 0.78)^54^. Based on our data, an acceptable internal consistency (α = 0.72) was found.

#### Physical activity (PA)

An adapted version of the validated International Physical Activity Questionnaire (IPAQ, Dutch long version) was used to estimate participants’ PA during the last seven days^55^. The questions regarding SB were left out, given that participants’ SB was assessed by another validated and more specific questionnaire (see below). The self-report IPAQ questionnaire includes 31 items to assesses PA in four domains: work-related, transport-related, domestic and garden, and other leisure-time PA (e.g., “How much time did you usually spend on one of those days doing moderate physical activities inside your home?”). All outcomes were expressed in hours/week. Standard scoring criteria were applied: only bouts of ten or more consecutive minutes were retained, non-relevant observations were excluded (e.g., answering in step counts instead of minutes), and PA levels higher than 960 min/day (i.e., 16 h/day) were excluded^56^. The IPAQ has fair to good psychometric properties^57^.

#### Sedentary behavior (SB)

SB was assessed by the Dutch version of the validated context-specific sedentary behavior questionnaire of Busschaert, De Bourdeaudhuij^58^. Three SB domains were assessed: work-related, transport-related, and leisure-time SB. Participants were asked to specify how much time they spent sitting/lying down during the last seven days (for weekdays and weekend days separately) within each SB domain (e.g., “How much time did you spend, on average, on a day during the last seven days on transportation from, to and during work?”). They were asked to fill in the number of days and the amount of time spent sitting/lying for several items/activities (e.g., TV watching, computer use, reading) within each of the three domains. For each item, a specific time interval could be chosen (e.g., 1 to 15 min, 15 to 30 min, 30 to 60 min, 1 to 2 h, etc.). Subsequently, midpoint values of each interval were calculated (e.g., 7.5 min, 22.5 min, 45 min, 90 min, etc.). As it was not mentioned in the protocol^58^ how the upper limit time intervals (i.e., “more than seven hours a day” and “more than eight hours a day”) for items/activities had to be interpreted, it was decided to consider these time intervals as values of 450 min and 510 min, respectively. For descriptive purposes, total SB (expressed in hours/week) was estimated by summing all midpoint values of the specific SB domains (for weekdays and weekend days separately) and was then calculated as follows: (self-reported total sedentary time on a weekday * 5) + (self-reported total sedentary time on a weekend day * 2). Consistent with the IPAQ scoring protocol approach, we decided to exclude participants with total SB levels higher than 960 min/day (i.e., 16 h/day) from the analysis. The context-specific sedentary behavior questionnaire has moderate to good psychometric properties^58^.

#### Dietary behavior

To measure participants’ intake of fruits and vegetables and to estimate their diet quality, a validated Food Frequency Questionnaire (FFQ) was used, containing 24 items questioning the intake of different food groups (e.g., fruits, vegetables, etc.) based on the national Food Based Dietary Guidelines^59^. This FFQ included questions about the frequency of intake (i.e., never, 1 to 3 days per month, 1 day per week, 2 to 4 days per week, 5 to 6 days per week and every day) as well as the (regular) portion size of each food group (e.g., “On the days you consumed fruit, how much fruit did you, on average, consume per day?”). Every question regarding the portion size included a small list of common standard measures (e.g., 1 tomato = 150 g). The consumed portion of each food group was calculated by multiplying the consumption per day by the specified portion size per day. In addition, to calculate the consumed amount of energy that each food group contains (i.e., kcal/day of each food group), the weighted average energy content of each food group was multiplied with the daily consumed portion. Total energy intake was calculated by computing and adding the kcal/day intake of all food groups. Finally, to adjust daily food group intakes for total energy intake, daily consumed portions of each food group were divided by the total kcal intake and multiplied by 1000 in order to have the daily intake in g per 1000 kcal. For the purpose of this study, only average daily intakes of fruits (g/day) (i.e., fresh, canned, frozen and dried fruit) and vegetables (g/day) (i.e., raw and cooked vegetables) were used in the statistical analyses. A total Dietary Quality Index (DQI) was calculated by summing the dietary diversity, dietary quality and dietary equilibrium, divided by three. Dietary diversity expresses the variation between the eight recommended food groups. Dietary quality expresses whether a person chooses for food groups from the ‘preference group’, the ‘moderation group’ or the ‘low nutritious group or energy dense group’. Dietary equilibrium expresses the balance of the food group intake. The dietary equilibrium is calculated by the sum of dietary adequacy and moderation. The total DQI ranges from 0–100; the higher, the better the diet quality. The used FFQ has acceptable psychometric properties^60^.

#### Sleep

Sleeping time was assessed by questions based on the so-called ‘health-related behavior’ part of the Health Behavior in School-Aged Children(HBSC)-questionnaire^61^. Participants were asked how many hours they slept on average per night during the week and during the weekend. A distinction between week and weekend is made as it can be assumed that people’s sleeping time may differ between the week and the weekend according to the burnout risk and/or recovery need they experience.

### Statistical analyses

All data were analyzed using R (R core Team, 2019; R Studio version 3.6.2). Prior to the analyses, all data were screened for outliers and data cleaning was performed. Representativeness of the sample was assessed by conducting two proportions z-tests comparing our sample against the population of Flemish secondary schoolteachers on sex, age, and education network^62^. Preliminary analyses checked if a two level model was recommended (i.e., participants clustered within schools) by inspecting the amount of variance explained by the school-cluster. After calculating intraclass correlation coefficients (ICCs), it was decided not to take the school-cluster into account, as low ICCs were found regarding the burnout risk and recovery need outcomes (i.e., emotional exhaustion: ICC = 0.0004, depersonalization: ICC = 0.009, personal accomplishment: ICC = 0.004; recovery need: ICC = 0.05). Multiple linear regression models were applied with the lm() function of the lme4-package in R^63^ to identify which lifestyle factors significantly predicted the scores on each burnout risk dimension and the score on recovery need. Since lifestyle factors could be assumed to be confounders or mediators of the relationship between another lifestyle factor and burnout, and because models should be adjusted for confounding lifestyle factors only and not for mediating lifestyle factors^64^, two kinds of models were applied. The first model was based on the assumption that the burnout-lifestyle relationship is mediated by the other lifestyle factors (i.e. step-1 models) and the second model was based on the assumption that the burnout-lifestyle relationship is confounded by the other lifestyle factors (i.e. step-2 models). This means that step-1 models included only one lifestyle factor (i.e., work-related PA, transport-related PA, domestic and garden PA, leisure-time PA; work-related SB, transport-related SB, leisure-time SB; daily intake of fruits, daily intake of vegetables, diet quality; sleeping time during the week and sleeping time during the weekend), in addition to sex, age, being a parent and weekly amount of teaching. In these step-1 models, it is thus assumed that the relationship between one lifestyle factor and the burnout risk or recovery need outcomes is mediated through the other lifestyle factors and, therefore, the model should not be adjusted for these other lifestyle factors^64^. In the step-2 models, all lifestyle factors were combined into one model adjusted for sex, age, being a parent and weekly amount of teaching. In this combined model, it is thus assumed that the relationship between one lifestyle factor and the burnout risk or recovery outcomes is confounded by the other lifestyle factors and, therefore, the model needs to be adjusted for the other lifestyle factors^64^. Multicollinearity was checked by interpreting the Variation Inflation Factor (VIF). The above-described approach was performed separately for each of the four outcome measures (i.e., emotional exhaustion, depersonalization, personal accomplishment and recovery need). *P* values < 0.05 were considered statistically significant.

### Ethical approval

Ethical approval was received from the Medical Ethics Committee of the University Hospital (UZ Brussel, Brussels, Belgium; B.U.N. 143201940533). Research was performed in accordance with relevant guidelines/regulations and all participants gave their informed consent prior to participating in the study.

## Results

The online questionnaire was completed by 2220 secondary schoolteachers. Of these initial 2220 participants, 1878 remained after applying the predefined exclusion criteria (i.e., not working in secondary education (n = 24), sick leave not due to burnout (n = 23), an adapted PA and/or dietary pattern (n = 175), missing values for IPAQ (n = 31), being an outlier for total PA (n = 58), being an outlier for total SB (n = 31)).

### Sample characteristics

The total study sample consisted of 1878 secondary schoolteachers (77.3% females) with a mean age of 41.1 ± 10.2 years and a mean BMI of 24.9 ± 4.6 kg/m^2^ (see Table 1).

**Table 1:** Study sample characteristics.

Sample characteristics (n = 1878)	Mean ± SD, %
Age (years)	41.1 ± 10.2
Sex (% of females)	77.3
Marital status (%)	
Single	12.9
Not married	5.4
Married	51.8
Living together	23.4
Divorced	6.0
Widowed	0.6
Being a parent (%)	72.3
Ethnicity (%)	
White, European	98.5
White, other	0.3
North-African	0.3
Middle Eastern	0.3
South Asian	0.1
Southeast Asian	0.1
Mixed	0.5
Highest diploma (%)	
Secondary school degree^a^	2.4
Post-secondary school degree	0.5
Bachelor’s degree	57.1
Master’s degree	38.7
PhD degree	1.3
Weekly amount of teaching (h/week)	18.9 ± 4.8
BMI (kg/m^2^)	24.9 ± 4.6
Total physical activity (h/week)	29.6 ± 22.8
Work-related physical activity (h/week)	10.9 ± 14.0
Transport-related physical activity (h/week)	4.3 ± 5.4
Domestic and garden physical activity (h/week)	10.0 ± 10.8
Leisure-time physical activity (h/week)	4.4 ± 6.4
Total sedentary behavior (h/week)	52.6 ± 18.7
Work-related sedentary behavior (h/week)	12.6 ± 7.7
Transport-related sedentary behavior (h/week)	7.0 ± 5.9
Leisure-time sedentary behavior (h/week)	21.6 ± 10.8
Sleeping time during week (h/night)	7.0 ± 0.9
Sleeping time during weekend (h/night)	8.1 ± 1.4
Daily intake of fruits (g/1000 kcal/day)	131.8 ± 105.9
Daily intake of vegetables (g/1000 kcal/day)	231.7 ± 133.8
Dietary Quality Index (%)	73.1 ± 10.0
Burnout risk (%)	25.5
High recovery need (%)	55.2

*M* mean, *SD* standard deviation, *BMI* body mass index.

^a^In Belgium, people with a secondary school degree are allowed to teach some subjects in technical and vocational secondary education (‘Diploma's die toelating geven tot een betrekking in het secundair onderwijs’, https://data-onderwijs.vlaanderen.be/bekwaamheidsbewijzen/lijst.aspx?s=diploma&niv=SO&g=).

### Representativeness of the sample

To assess its representativeness, the sample of this study was compared to the general secondary schoolteacher population in Flanders^62^ (see Appendix A—Table 1). Two proportion z-tests indicated that our study sample consisted of more females than the general population (77.3 vs. 64.9%; *p* < 0.001), less teachers in the age group 60 + (3.3% vs. 5.8%; *p* < 0.001), more teachers teaching in Flemish community schools (51.2% vs. 22.5%; *p* < 0.001) and less teachers teaching in subsidized free schools (45.5% vs. 68.0%; *p* < 0.001) and in subsidized public schools (3.4% vs. 9.4%; *p* < 0.001).

### Physical activity (PA)

The step-1 and step-2 models showed that *emotional exhaustion* (see Table 2—part 1) was significantly and positively associated with domestic and garden PA (step-1 model: *p* = 0.016; step-2 model: *p* = 0.010). For example, the step-2 model showed that one hour per week more domestic and garden PA was associated with a mean of 0.007 units higher score on *emotional exhaustion.* Regarding leisure-time PA, only the step-1 model showed a significant and negative association with *emotional exhaustion* (*p* = 0.39) demonstrating that one hour per week more leisure-time PA is associated with a mean of 0.009 units higher score on *emotional exhaustion*. The step-2 model showed no significant associations of work-related PA, transport-related PA and leisure-time PA with *emotional exhaustion*. *Depersonalization* (see Table 2—part 1) was positively associated with leisure-time PA both in the step-1 and step-2 models (step-1 model: *p* = 0.006; step-2 model: *p* = 0.002), whereas positive associations with work-related PA (*p* = 0.037) and domestic and garden PA (*p* = 0.025) were only found in the respective step-1 models. No significant associations with *depersonalization* were found for transport-related PA (both models), and work-related PA and domestic and garden PA in the step-2 model. Regarding *personal accomplishment* (see Table 2—part 2), only a positive association with work-related PA in the step-2 model was found (*p* = 0.047). Work-related PA (step-1 model), transport-related PA, domestic and garden PA and leisure-time PA did not show significant associations with *personal accomplishment*. *Recovery need* (see Table 2—part 2) was found to be positively associated with domestic and garden PA in both the step-1 and step-2 models (step-1 model: *p* = 0.040; step-2 model: *p* = 0.039), while negative associations were found for leisure-time PA (step-1 model: *p* = 0.004; step-2 model: *p* = 0.003). No significant associations with *recovery need* were observed for work- and transport-related PA.

**Table 2:** Physical activity, sedentary behavior, dietary behavior and sleep related correlates of burnout risk and/or recovery need in secondary schoolteachers.

Part 1								
	Emotional exhaustion				Depersonalization			
	Estimates ± SE		*p* values		Estimates ± SE		*p* values	
	Step-1 model	Step-2 model	Step-1 model	Step-2 model	Step-1 model	Step-2 model	Step-1 model	**Step-2 model**
Physical activity (h/week)								
Work-related PA	0.0008 ± 0.0020	0.0002 ± 0.0021	0.679	0.923	**0.003 ± 0.001**	0.002 ± 0.001	**0.037**	0.175
Transport-related PA	0.0002 ± 0.0050	0.006 ± 0.005	0.965	0.278	0.005 ± 0.003	0.003 ± 0.004	0.156	0.401
Domestic and garden PA	**0.006 ± 0.003**	**0.007 ± 0.003**	**0.016**	**0.009**	**0.004 ± 0.002**	0.003 ± 0.002	**0.025**	0.066
Leisure-time PA	**− 0.009 ± 0.004**	− 0.008 ± 0.004	**0.039**	0.086	**0.008 ± 0.003**	**0.009 ± 0.003**	**0.006**	**0.002**
Sedentary behavior (h/week)								
Work-related SB	**0.014 ± 0.003**	**0.013 ± 0.004**	** < 0.001**	** < 0.001**	**0.008 ± 0.002**	**0.009 ± 0.002**	**0.001**	**0.001**
Transport-related SB	0.00010 ± 0.00008	− 0.00006 ± 0.00008	0.197	0.472	0.00008 ± 0.00005	0.00002 ± 0.00006	0.139	0.724
Leisure-time SB	− 0.0003 ± 0.0018	− 0.001 ± 0.002	0.883	0.575	0.002 ± 0.001	0.001 ± 0.001	0.068	0.323
Dietary behavior								
Fruit intake (g/day/1000 kcal)	**− 0.0007 ± 0.0003**	**− 0.0006 ± 0.0003**	**0.009**	**0.039**	**− 0.0005 ± 0.0002**	**− 0.0005 ± 0.0002**	**0.005**	**0.014**
Vegetable intake (g/day/1000 kcal)	− 0.00001 ± 0.00021	0.0003 ± 0.0002	0.944	0.178	− 0.0001 ± 0.0001	0.00005 ± 0.00016	0.478	0.729
Diet quality	**− 0.0086 ± 0.0027**	− 0.0045 ± 0.0031	**0.002**	0.145	**− 0.0043 ± 0.0018**	− 0.0006 ± 0.0022	**0.017**	0.768
Sleep (h/night)								
Sleeping time during week	**− 0.16 ± 0.03**	**− 0.20 ± 0.03**	** < 0.001**	** < 0.001**	− 0.03 ± 0.02	− 0.03 ± 0.02	0.214	0.240
Sleeping time during weekend	**0.05 ± 0.02**	**0.1 ± 0.02**	**0.016**	** < 0.001**	0.01 ± 0.01	0.03 ± 0.02	0.454	0.088

Part 2								
	Personal accomplishment				Recovery need			
	Estimates ± SE		*p* values		Estimates ± SE		*p* values	
	Step-1 model	Step-2 model	Step-1 model	Step-2 model	Step-1 model	Step-2 model	Step-1 model	Step-2 model
Physical activity (h/week)								
Work-related PA	0.003 ± 0.001	**0.003 ± 0.002**	0.067	**0.047**	− 0.001 ± 0.003	− 0.002 ± 0.003	0.696	0.476
Transport-related PA	0.002 ± 0.004	− 0.003 ± 0.004	0.628	0.502	− 0.0002 ± 0.0072	0.013 ± 0.008	0.978	0.098
Domestic and garden PA	0.002 ± 0.002	0.001 ± 0.002	0.286	0.614	**0.007 ± 0.004**	**0.008 ± 0.004**	**0.040**	**0.039**
Leisure-time PA	− 0.002 ± 0.003	− 0.004 ± 0.003	0.621	0.258	**− 0.017 ± 0.006**	**− 0.019 ± 0.007**	**0.004**	**0.003**
Sedentary behavior (h/week)								
Work-related SB	0.005 ± 0.003	**0.006 ± 0.003**	0.063	**0.034**	**0.014 ± 0.005**	**0.011 ± 0.005**	**0.004**	**0.035**
Transport-related SB	− 0.00003 ± 0.00006	0.000002 ± 0.000063	0.612	0.979	**0.0003 ± 0.0001**	0.0002 ± 0.0001	**0.012**	0.150
Leisure-time SB	0.0006 ± 0.0013	0.001 ± 0.001	0.648	0.479	− 0.002 ± 0.003	− 0.003 ± 0.003	0.520	0.202
Dietary behavior								
Fruit intake (g/day/kcal)	**0.0005 ± 0.0002**	0.0002 ± 0.0002	**0.005**	0.294	− 0.0007 ± 0.0004	− 0.0003 ± 0.0004	0.053	0.507
Vegetable intake (g/day/kcal)	**0.0004 ± 0.0002**	0.0003 ± 0.0002	**0.004**	0.062	− 0.00006 ± 0.00029	− 0.00002 ± 0.00033	0.841	0.945
Diet quality	**0.0059 ± 0.0020**	0.0032 ± 0.0024	**0.003**	0.176	**− 0.008 ± 0.004**	− 0.0040 ± 0.0045	**0.033**	0.381
Sleep (h/night)								
Sleeping time during week	0.02 ± 0.02	0.03 ± 0.02	0.436	0.246	**− 0.21 ± 0.04**	**− 0.22 ± 0.05**	** < 0.001**	** < 0.001**
Sleeping time during weekend	− 0.01 ± 0.01	− 0.03 ± 0.02	0.734	0.138	0.02 ± 0.03	**0.09 ± 0.03**	0.416	**0.007**

All models were adjusted for sex, age, being a parent and weekly amount of teaching (in h/week).

Step-1 model = Lifestyle factors separately.

Step-2 model = Fully-adjusted model including all lifestyle factors.

Emotional exhaustion, depersonalization and personal accomplishment: scale ranging from 0 to 6 (Emotional exhaustion: higher scores = feeling more emotionally exhausted, depersonalization: higher scores = feeling more cynical about their job, personal accomplishment: higher scores = feeling more competent at work).

Recovery need: scale ranging from 0 to 5 (a score ≥ 3 = having a ‘high need for recovery’).

*SE* standard error, *PA* physical activity, *SB* sedentary behavior.

Significant *p* values (i.e., < 0.05) in bold font.

### Sedentary behavior (SB)

The step-1 and step-2 models showed that *emotional exhaustion* (see Table 2—part 1) was positively associated with work-related SB (step-1 and step-2 model: *p* < 0.001). The step-2 model, for example, showed that one hour per week more SB at work is associated with a mean of 0.013 units higher score on *emotional exhaustion*. Transport-related SB and leisure-time SB did not show significant associations with *emotional exhaustion*. *Depersonalization* (see Table 2—part 1) was found to be positively associated with work-related SB both in the step-1 and step-2 models ((step-1 and step-2 model: *p* = 0.001), whereas no significant associations were found for transport-related SB and leisure-time SB. Regarding *personal accomplishment* (see Table 2—part 2), only the step-2 model showed a positive association for work-related SB (*p* = 0.034). Work-related SB (step 1-model), transport-related SB and leisure-time SB (step-1 and step-2 models) were not significantly associated with *personal accomplishment*. *Recovery need* (see Table 2—part 2) was positively associated with work-related SB both in the step-1 and step-2 models (step-1 model: *p* = 0.004; step-2 model: *p* = 0.035). Regarding transport-related SB, only the step-1 model showed a positive association with *recovery need* (*p* = 0.012). No significant associations with *recovery need* were observed for transport-related (step-2 model) and leisure-time SB.

### Dietary behavior

Both in the step-1 and step-2 models, *emotional exhaustion* (see Table 2—part 1) was negatively associated with fruit intake (step-1 model: *p* = 0.009; step-2 model: *p* = 0.039). For example, the step-2 model found that one gram/day/1000 kcal higher fruit intake is associated with a mean of 0.0006 units lower score on *emotional exhaustion*. *Emotional exhaustion* showed only a significant association with diet quality in the step-1 model (*p* = 0.002). Vegetable intake did not show a significant association with *emotional exhaustion*. The step-1 and step-2 models for *depersonalization* (see Table 2—part 1) showed a negative association with fruit intake (step-1 model: *p* = 0.005; step-2 model: *p* = 0.014). Regarding *depersonalization* and diet quality, only the step-1 model showed a negative association (*p* = 0.037). No significant association with *depersonalization* was observed for vegetable intake nor for diet quality (step-1 model). *Personal accomplishment* (see Table 2—part 2) was positively associated with fruit intake (*p* = 0.005), vegetable intake (*p* = 0.004) and diet quality (*p* = 0.010) in the step-1 models. The step-2 models with fruit intake, vegetable intake and diet quality were not significantly associated with *personal accomplishment*. *Recovery need* (see Table 2—part 2) only showed a significant association with diet quality (*p* = 0.033) in the step-1 model.

### Sleep

The step-1 and step-2 models showed that *emotional exhaustion* (see Table 2—part 1) was negatively associated with sleeping time per night during the week (step-1 and step-2 models: *p* < 0.001). For example, the step-2 model found that one more hour of sleep per night during the week is associated with a mean of 0.2 units lower score on *emotional exhaustion*. Both step-1 and step-2 models showed positive associations between *emotional exhaustion* and sleeping time per night during the weekend (step-1 model: *p* = 0.016; step-2 model: *p* < 0.001), demonstrating that one more hour of sleep per night during the weekend is associated with a mean of 0.1 units higher score on *emotional exhaustion*. Sleep was not significantly associated with *depersonalization* (see Table 2—part 1) nor with *personal accomplishment* (see Table 2—part 2), whereas sleeping time per night during the week was significantly and negatively associated with *recovery need* (see Table 2—part 2) both in the step-1 and step-2 models (step-1 and step-2 model: *p* < 0.001). Regarding sleeping time per night during the weekend, only the step-2 model showed a positive association with *recovery need* (*p* = 0.007). The step-1 model did not show a significant association between *recovery need* and sleeping time per night during the weekend.

## Discussion

The purpose of this study was to gain insight into the associations of PA, SB, dietary behavior and sleep with burnout risk and recovery need in Flemish secondary schoolteachers. The relevance of these research questions are illustrated by our observation that 25.5% of our study participants reported risk of burnout, which clearly exceeds the 13% of the general population (in Flanders) reporting burnout symptoms^8^, and that 55.2% of the teachers showed a high need for recovery. These results are worrying, especially because all self-reported data were collected in September–October 2019 when the school year 2019–2020 had only just begun. As hypothesized, associations between some lifestyle factors and burnout dimensions, as well as recovery need, were found. However, not all lifestyle factors were associated with burnout risk and recovery need, and the strength of associations differed. In line with our hypothesis, our results demonstrated that emotional exhaustion was significantly associated with work-related SB (positive association, +), fruit intake (negative association, −) and sleeping time per night during the week (−), depersonalization showed significant associations with work-related SB and fruit intake (−), whereas leisure-time PA (−), work-related SB (+) and sleeping time per night during the week (−) were found to be significantly associated with recovery need. Contrary to our hypothesis, we found that emotional exhaustion was significantly associated with domestic and garden PA (+) and sleeping time during the weekend (+), depersonalization showed a significant association with leisure-time PA (+), whereas domestic and garden PA (+) were significantly associated with recovery need. Work-related PA, transport-related PA, transport-related SB, leisure-time SB, vegetable intake and diet quality were not associated with the burnout dimensions, nor with recovery need (in both step-1 and step-2 models).

The first part of the discussion will only focus on the predictors showing significant associations in both step-1 and step-2 models.

### Physical activity (PA), burnout risk and recovery need

Teachers with higher levels of domestic and garden PA were more emotionally exhausted and showed a higher need for recovery. A possible reason for these positive associations may be the fact that activities being part of domestic and garden PA (i.e., cleaning the house, ironing, cooking, weeding, mowing the lawn, …) feel more compulsory than other kind of physical activities, yielding more extrinsic motivation and more negative feelings such as anxiety and tension^65^. In contrast, a recent study by de Vries and Bakker^30^ did not find an association between burnout risk and domestic and garden PA. It should be mentioned that the research by de Vries and Bakker^30^ was conducted in a heterogenous sample of full-time workers and burnout risk was measured by the Burnout Assessment Tool (BAT)^66^. The latter study also indicated a positive association between transport-related PA and burnout risk, which is contradictory to our findings. Further, the present study found that teachers being more physically active during leisure-time showed a lower need for recovery, whereas higher scores for depersonalization were found. Previous research has shown that leisure-time activities, which are often voluntary and chosen for enjoyment, indeed aid recovery^30,67^. Moreover, a study by Isoard-Gautheur, Ginoux^67^ found that being more intrinsically motivated to be physically active reinforces the positive moderating role of PA in the stressor-burnout relationship. People being more intrinsically motivated for PA experience more pleasure and are more absorbed in doing the particular activity^30,65,67–69^, which helps to distract them from work-related problems and to replenish more easily their personal resources to recover from and cope with job demands^30,32,65,67–69^. The positive association between leisure-time PA and depersonalization may be due to the fact that people who take more distance from their work while being more cynical about their job, focus more on their off-job activities, including leisure-time PA.

### Sedentary behavior (SB), burnout risk and recovery need

Teachers with higher levels of SB at work showed more emotional exhaustion, more depersonalization and more recovery need, which is in line with the findings of two previous studies demonstrating a positive association between burnout risk and SB^70,71^. It should be mentioned though that both of these studies assessed SB by means of the 4-level Saltin Grimby Physical Activity Level Scale^72^, of which the lowest level reflects SB, not allowing any further distinctions in SB domains. It is interesting that especially SB at work predicted burnout risk and recovery need, while transport-related SB and leisure-time SB did not (robustly). In analogy with domestic and garden PA, the compulsory nature of work-related SB compared to SB in the other domains may explain why SB at work predicted burnout risk. Reversely, higher levels of SB at work may also be the result of the exhaustion teachers experience. Another possible explanation is that teachers who are at higher burnout risk need to invest extra effort to perform well at work/uphold their work performance^73^. As such, performing well at work is of high priority for teachers who are at burnout risk, which refrains them from taking frequent standing breaks because (they believe) these interfere with productivity^74^.

### Dietary behavior, burnout risk and recovery need

Teachers with lower fruit intake per day showed higher scores for emotional exhaustion and depersonalization. These results are in accordance with a recent study by Penttinen, Virtanen^42^ showing higher fruit intake and more frequent consumption of so-called “healthy food” items to be associated with lower burnout scores. Despite the fact that the latter study also found higher vegetable intake to be related to lower burnout scores^42^, the present study only found a positive association between vegetable intake and personal accomplishment in the step-1 model. In contrast to our study, however, the study by Penttinen, Virtanen^42^ made a distinction between different kinds of vegetables (i.e., fresh vegetables, cooked vegetables, root vegetables and legumes), showing only significant negative associations for fresh and cooked vegetables with burnout scores. Our study did not find any associations between dietary behavior and recovery need. To the best of our knowledge, this is the first study assessing the relationship between dietary behavior and recovery need, precluding any comparison with previous research. The observed negative associations between fruit intake and the burnout dimensions may be explained by the potential of fruits and vegetables to promote higher levels of optimism and self-efficacy, to reduce psychological distress and to protect against depressive symptoms^35^. In addition, research showed that a sufficient fruit and vegetable intake is crucial for maintaining cognitive functions^75^. As mentioned in the study by Penttinen et al.^42^, the association between a healthy diet and burnout risk might be also explained by the following two mechanisms: the anti-inflammatory mechanism(s) and the gut-brain axis. First, it is known that a healthy diet may have anti-inflammatory effects in the human body^76^. Moreover, a systematic review by Tolkien, Bradburn^76^ about depression (which shares similar symptoms with burnout) concluded that an association exists between a pro-inflammatory diet (i.e., for example a diet high in red meats, refined grains, saturated and trans-fatty acids) and risk of depression^76^. A second mechanism that may explain the healthy diet-burnout risk relationship concerns the effect of food substances on the gut microbiota, since it is shown that gut microbiota can directly affect brain function via the gut-brain axis, and thereby may affect mood as well as feelings of anxiety and depression^77,78^.

### Sleep, burnout risk and recovery need

More emotional exhaustion and higher recovery need were associated with less sleep per night during the week. In contrast, teachers with higher scores for emotional exhaustion showed more sleep per night during the weekend. It can be assumed that people with high scores of emotional exhaustion sleep more hours per night during the weekend in order to recover from the work week and the limited sleeping time during week nights. Another possible explanation is that people with high burnout risk are less able to control their thoughts about work-related issues, especially during the week, which may result in a decreased ability to fall asleep. Research indeed showed that perseverative cognition (i.e., “repeated or chronic activation of the cognitive representation of one or more psychological stressors”^79^) can be an important underlying mechanism in the work-related stress and sleep quality relationship^80^. A study by Cropley, Dijk^81^, on the other hand, found that high job strain teachers indeed ruminated more about work-related issues during bedtime and reported poorer sleep quality, but no difference in total sleep time compared to low job strain teachers was found. Our results are in line with previous research regarding sleep and burnout risk, showing sleep deprivation and poor(er) sleep quality to be related with burnout risk^44,46^. However, a recent review regarding sleep and burnout risk concluded that despite the fact that cross-sectional research indeed generally indicates a negative relationship between sleep quality and burnout risk, the findings of a few longitudinal studies are very heterogeneous in this respect^46^. As sleep deprivation has been associated with several negative mental health consequences, which are similar to symptoms of burnout, such as mood changes, fatigue, increased sleepiness, excess mental distress, depressive symptoms, etc.^82^, it is important to further investigate and unravel the role of sleep in burnout prevention.

### Statistical interpretations

Some lifestyle factors were only found to be significant in the step-1 or step-2 models. The fact that some predictors were found to be significant in the step-1 models, but not in the step-2 models, may be explained by three possible mechanisms. In the step-2 models, all lifestyle factors were included, and thus when *p* values increase, which means a decrease of the magnitude of the relationship between the independent and dependent variables, one (or more) of the added lifestyle factors may serve either as (a) confounder(s), as (a) mediator(s) or as (a) suppressor(s)^83–85^. The fact that some predictors were found to be significant in the step-2 models only, may be explained by another form of suppressor variables. These are defined by Conger^86^ as variables increasing the predictive validity of another variable (or set of variables) by its inclusion in a regression equation. Lastly, when a lifestyle factor serving as a collider (i.e., a common effect of the independent and dependent variable) was included in the model, a spurious association between the independent and dependent variables may exist, as controlling for colliders may create the illusion of a direct relationship between the independent and dependent variable^87^. As several mechanisms may cause different results between step-1 and step-2 models, the consistent associations found in both models were considered most reliable. Future longitudinal studies are required to allow for a correct temporal identification of confounding, mediating and collider variables and for a stronger causal interpretation of the observed associations.

An important note is that the effect sizes of all observed associations were rather small. This may be explained by the fact that lifestyle is just one of the many factors contributing to burnout (e.g., environmental risk factors: work overload, daylight, lack of control; individual risk factors: personality features such as neuroticism, extraversion, conscientiousness^88^). However, regarding PA for example, a recent systematic review, also including experimental studies, found much larger effect sizes with reductions in burnout risk (between 6.9 and 41.3%) induced by increased PA across different contexts^29^. It is important to mention, however, that the majority of the studies included in the latter review were conducted in employees showing high levels of burnout risk, which may partially explain the large(r) effect sizes. Also, none of those studies were conducted in a teaching population, but rather among white-collar workers. It was found that positive effects of leisure-time PA on burnout risk may depend on employees’ PA level at work (i.e., the ‘physical activity paradox’)^30^. Although teaching is not considered as a typical physically demanding job, a study by PY and Chow^89^ found that teachers are engaging in activity levels comparable with blue-collar workers. Furthermore, it is shown that Flemish secondary schoolteachers are more physically active compared to the general population in Flanders (i.e., 66.2% vs. 49.0% meeting the recommendation of 150 min MVPA per week)^90^. Regarding the clinical relevance of these effect sizes, our results regarding fruit intake for example shows that when consuming 100 g/1000 kcal more fruits per day, a lower score on emotional exhaustion with 0.06 scale-points is observed. As the average daily fruit intake of our sample is already 131.8 ± 105.9 g/1000 kcal/day, the potential to increase this intake in order to have relevant changes in burnout risk is limited. The same conclusions can be made regarding the other lifestyle factors. Therefore, it might be that rather a combined effect of lifestyle factors, instead of a single lifestyle factor, will have more clinically relevant effects on burnout risk. Nevertheless, despite the low effect sizes, the results of the present study indicate once again the importance of investing in the adherence to a healthy lifestyle.

### Strengths and limitations

The main strength of our study is that it is the first one assessing the association between burnout risk, recovery need and lifestyle, using a holistic approach (i.e., multiple lifestyle factors). In addition to this holistic approach, it is also the first study making a distinction between different PA and SB domains, which provides more insight into which type of contextual PA and SB may be effective in view of reducing and/or preventing burnout risk and recovery need. Third, both step-1 and step-2 models were analyzed to take into account mediating/confounding roles of lifestyle factors investigating their relationship with burnout risk and recovery need. A final strength concerns the large sample of secondary schoolteachers included in the present study. It should be mentioned though that our representativeness analysis showed that our study sample consisted of more females, more teachers aged between 30–39 years and less teachers being more than 50 years old as compared to the general population of Flemish schoolteachers in Flanders. As females are more likely to report burnout symptoms^91^, and as younger employees mostly experience more burnout than older employees^92^, the burnout risk and recovery need scores reported in the present study may be somewhat higher than in the actual secondary school teacher population. To counter possible effects of sex and age, alongside being a parent and weekly amount of teaching, we adjusted the models for these possible confounders. Further, although validated questionnaires were used in this study, the lifestyle factors of interest (i.e., PA, SB, dietary behavior and sleep) were measured subjectively. As our cross-sectional data collection was solely based on self-report, social desirability and recall bias may have been present, resulting in possible over- and underestimations, depending on the outcome measure. Nevertheless, we expect the abovementioned over- and underestimations to have had limited influence on the examined cross-sectional lifestyle-burnout risk/recovery need relationship, as we expect these to be systematic rather than unsystematic errors. Further, due to the compositional nature of daily activity data (PA, SB, sleep), future research may benefit from using a 24-h approach when measuring these behaviors^93^. Another point to note is that only (secondary school) teachers were included in this particular study, so we should be cautious in view of generalizing our results to other teachers, populations or occupations. Lastly, due to the cross-sectional design of this study, no conclusions regarding the causal relationship between lifestyle and burnout risk/recovery need can be made. For example, as it is already shown that people with burnout symptoms may be more vulnerable to emotional and uncontrolled eating behavior^94^, the association between dietary behavior and burnout risk may be bi-directional. The same applies for sleep, as a study by Armon, Shirom^95^ showed that burnout and insomnia recursively predict each other’s development. In order to get more insight into the (causal) relationship between multiple lifestyle factors, and burnout risk and recovery need in a teaching population, longitudinal and experimental research on this topic is warranted.

### Practical implications

Based on our results, a healthy lifestyle in secondary schoolteachers should be promoted and facilitated. To do so, most likely, interventions at the societal, school and individual level are needed. First of all, efforts should be undertaken to reduce teachers' work-related stressors, as it has been shown that job stressors do not only contribute to burnout, but also to an unhealthier lifestyle (e.g., Mutz, Abdel Hadi^96^). Additionally, schools could provide fruit for their teaching staff during working hours, design the environment in a way that teachers are encouraged to limit sitting time during work and teachers could be informed about the benefits of a healthy lifestyle.

## Conclusion

Domestic and garden PA, leisure-time PA, work-related SB, fruit intake and sleep may be important lifestyle factors to address when it comes to preventing and/or managing burnout risk and recovery need. Despite the low effect sizes and cross-sectional design, education networks and schools might benefit from promoting and facilitating a healthy lifestyle in secondary schoolteachers.

### Supplementary Information

Supplementary Information.

## Data Availability

Data are available upon reasonable request via the corresponding author.
