# Author Correction: Associations of lifestyle with burnout risk and recovery need in Flemish secondary schoolteachers: a cross-sectional study

**DOI:** 10.1038/s41598-024-58312-3

**Published:** 2024-04-09

**Authors:** Yanni Verhavert, Tom Deliens, Jelle Van Cauwenberg, Elke Van Hoof, Christophe Matthys, Juriena de Vries, Peter Clarys, Kristine De Martelaer, Evert Zinzen

**Affiliations:** 1 Department of Movement and Sport Sciences, Vrije Universiteit Brussel, Pleinlaan 2, 1050 Brussels, Belgium; 2 Department of Public Health and Primary Care, Faculty of Medicine and Health Sciences, Ghent University, Corneel Heymanslaan 10, 9000 Ghent, Belgium; 3 Research Foundation – Flanders (FWO), Leuvenseweg 38, 1000 Brussels, Belgium; 4 Brussels, Belgium; 5 Department of Endocrinology, University Hospitals Leuven, Herestraat 49, 3000 Leuven, Belgium; 6 Clinical and Experimental Endocrinology, Department of Chronic Diseases and Metabolism, KU Leuven, Herestraat 49, 3000 Leuven, Belgium; 7 Department of Health, Medical and Neuropyschology, University of Leiden, Wassenaarseweg 52, 2333 AK Leiden, The Netherlands

Correction to: *Scientifc Reports* 10.1038/s41598-024-53044-w, published online 08 February 2024

Peter Clarys was omitted from the author list in the original version of this Article.

The Author Contributions section now reads:

Contribution statement: Study design (YV, TD, KDM, EZ, EVH), data collection (YV), data analysis (YV, TD, JVC), methodology (YV, TD, KDM, EZ, EVH), interpretation of the results (YV, TD, JVC, EVH, CM, JDV, PC, KDM, EZ), writing—original draft (YV), writing—reviewing & editing (YV, TD, JVC, EVH, CM, JDV, PC, KDM, EZ). YV, TD, KDM, EZ, EVH verified the initial dataset. YV, TD and JVC take responsibility for the analysis.

The original Article and the Supplementary Information has been corrected.

### Supplementary Information

Supplementary Information.

