# Supplementary material for: Author Correction: Associations of lifestyle with burnout risk and recovery need in Flemish secondary schoolteachers: a cross-sectional study
Source: Sci Rep. 2024 Apr 9;14:8274. doi: 10.1038/s41598-024-58312-3 (PMC11004009; doi:10.1038/s41598-024-58312-3)
Supplement: Supplementary file 1 — Supplementary Information. [file 41598_2024_58312_MOESM1_ESM.pdf]

Associations of lifestyle with burnout risk and recovery need in Flemish secondary schoolteachers: a cross-sectional study

Verhavert Yanni[1*,] Deliens Tom[1], Van Cauwenberg Jelle[2,3], Van Hoof Elke[4], Matthys Christophe[5,6], de Vries Juriena[7], De Martelaer Kristine[1], Zinzen Evert[1]

Appendix A

Table 1. Representativeness of the study sample compared to the general secondary teacher population in Flanders

			**X²**	**p-value**
	**Study sample (n = 1,878)**	**Population (n = 77,802)**		
**Sex (n (%))**				
Males	427 (22.7%)	27,300 (35.1%)	123.31	<.001
Females	1451 (77.3%)	50,502 (64.9%)	121.31	<.001
	**Sample (n = 1,873)[$]**	**Population (n = 77,802)**		
**Age range (years) (n**				
**(%))**	300 (16.0%)	11,508 (14.8%)	2.18	.14
20-29	586 (31.3%)	22,844 (29.4%)	3.26	.07
30-39	523 (27.9%)	20,876 (26.8%)	1.11	.29
40-49	402 (21.5%)	18,071 (23.2%)	3.20	.07
50-59	62 (3.3%)	4,503 (5.8%)	20.79	<.001
+60				
	**Sample (n = 1,595)[$]**	**Population (n = 77,802)**		
**Education network (n (%))**				
Flemish community schools	816 (51.0%)	17,471 (22.5%)	726.42	<.001
Subsidized free schools	724 (45.2%)	52,937 (68.0%)	365.97	<.001
Subsidized public schools	55 (3.4%)	7,349 (9.4%)	66.49	<.001

[$]Sample sizes may differ from the initial 1,878 due to missing data.
